# Detecting the Site of Phosphorylation in Phosphopeptides Without Loss of Phosphate Group Using MALDI TOF Mass Spectrometry

**DOI:** 10.4137/aci.s497

**Published:** 2008-02-26

**Authors:** Medicharla V. Jagannadham, Ramakrishnan Nagaraj

**Affiliations:** Centre for Cellular and Molecular Biology, Uppal Road, Hyderabad 500 007, India

**Keywords:** phosphopeptides, mass spectrometry, positive ion, neutral loss, linear mode, reflector mode

## Abstract

Phosphopeptides with one and four phosphate groups were characterized by MALDI mass spectrometry. The molecular ion of monophosphopeptide could be detected both as positive and negative ions by MALDI TOF with delayed extraction (DE) and in the reflector mode. The tetraphospho peptide could be detected in linear mode. When MS/MS spectra of the monophospho peptides were obtained in a MALDI TOF TOF instrument by CID, b and y ions with the intact phosphate group were observed, in addition the b and y ions without the phosphate group. Our study indicates that it is possible to detect phosphorylated peptides with out the loss of phosphate group by MALDI TOF as well as MALDI TOF TOF instruments with delayed extraction and in the reflector mode.

## Introduction

Post-translational modification (PTM) of proteins by phosphorylation is extensively observed in biological systems. It is a dynamic process effecting the folding and function of proteins (Tholey et al. 1999a; Moran et al. 2006). This process influences several cellular functions such as metabolic maintenance, cell division and signal transduction (Stock et al. 2000; Mumby and Brekenn, 2005; Morandell et al. 2006). The large number of functions that are influenced by the phosphorylation indicates the diverse role played by phosphorylation. Several amino acids in proteins can be phosphorylated. These include the common and well-known O-phosphates of serine, threonine and tyrosine residues and also unusual amino acids such as hydroxyproline, hydroxylysine (Sickmann and Meyer, 2001). Some lesser-known phosphorylations on amino acids such as histidine, lysine (N-phosphates), cysteine (S-phosphate), aspartic, glutamic acids (acyl-phosphates) were also identified in some proteins (Yan et al. 1998; Mclachilin and Chait, 2001; Reinders and Sickmann, 2005). In particular, phosphorylation of threonine, serine are known to play key roles in the regulation of the activities of the proteins involved in signal transduction in cells and is also important in the virulence mechanism of some pathogenic bacteria (Walburger et al. 2004; Papavinasa sundaram et al. 2005; Peris et al. 2005).

Characterization of these wide varieties of phosphorylations demands the development of a large number of strategies and methodologies. In tune with the requirements, several strategies are being developed (Yan et al. 1998; Kalume et al. 2003; Hjerrild and Gammeltoft, 2006; Moran et al. 2006; Ptacek and Snyder, 2006; Wang et al. 2006).

Mass spectrometry is a powerful tool due its capability to analyze complex mixtures. It is versatile, easy to use, produces spectra with high mass accuracies and great sensitivity. It is being extensively used for the characterization of PTMs. MALDI TOF and ESI mass spectrometry have been used extensively to identify PTMs (Jonscher et al. 1997; Larsen and Roepstorff, 2000; Lee et al. 2001; Salih, 2005; Loyet et al. 2005; Wang et al. 2006; Hardouin et al. 2006; Molle et al. 2006). Some of these studies demonstrated neutral loss of phosphate group, HPO_3_ or H_3_PO_4_ from the peptides detected by a reduction of 80 or 98 Da in the mass, due to metastable decomposition during MALDI TOF experiments in the reflector mode of acquisition of the spectrum (Annan and Carr, 1996; Neubauer and Mann, 1999; Kinumi et al. 2000). It was also shown that phosphoserine and phosphothreonine-containing peptides display significant intensity signals of the ions originating from the neutral loss of phosphoric acid, via gas phase β-elimination of the phosphate group (Neubauer and Mann, 1999). It was observed that all the y and b daughter ions containing these phosphoresidues were associated with this loss of phosphoric acid (Paizs and Suhai, 2005). In this process, the phosphoserine and the phosphothreonine residues are converted to dehydroalanine and dehydroamino-2-butyric acid respectively (Tholey et al. 1999b). Precursor ion scan and neutral ion scan methods can also be used to identify the phosphorylations in proteins and peptides (Wang et al. 2006; Domon and Abersold, 2006).

In source decay (ISD) and post source decay (PSD) have been used for the identification of the phosphorylation sites in peptides (Hoffmann et al. 1999; Kinumi et al. 2000; Chaurand et al. 1999; Lennon and Walsh, 1999; Talbo et al. 2001). However, lack of spectral information in the mass range 100–700 due to the interference of matrix cluster ions in the ISD spectra limits the detection of phosphorylation sites in this mass region. PSD cannot be used routinely, due to poor fragmentation of the peptides.

Phosphopeptide identification from the tryptic digests of proteins was successfully carried out with the help of MALDI peptide mapping before and after phosphatase treatment that results in a mass shift of 80 Da due to the removal of phosphate moiety (Liao et al. 1994; Wang et al. 1993; Larsen et al. 2001; Molle et al. 2006). MALDI ion trap MS was also used for the identification of phosphopeptides from protein digests (Quin and Chait, 1997). MALDI TOFMS has been used extensively for the detection of phosphopeptides. With delayed extraction of ions, it was shown to be a better choice as compared to the continuous extraction of ions (Vestal et al. 1995). Recent developments in the area of mass spectrometry, particularly with the availability of instrument such as MALDI TOF TOF could aid in not only detecting the phosphopeptides, but also mapping the site of phosphorylation.

In the present study, we show that mono phosphorylated peptides can be detected without loss of the phosphate group by MALDI mass spectrometry with delayed extraction, in the reflector mode. A tetra-phosphorylated peptide could be detected without loss of the phosphate groups in the linear mode. We have also observed that it is possible to detect peptide with the phosphate group intact when fragmented by CID in a MALDI TOF TOF instrument.

## Materials and Methods

α-cyano-4-hydroxy cinnamic acid (HCCA), monophosphopeptide, FQ[pS]EEQQQTEDELQDK(F16Kp),tetraphosphopeptide,RELEENVPGEIVE[p S]L[pS][pS][pS]EESITR(R25R) of casein digest, enzymatically dephosphorylated casein were purchased from sigma chemical co(St.Louis, MO, USA). Peptides Ac-EGTHSFDG-am (E8G) and its phosphorylated form were synthesized using Fmoc chemistry and purified by reverse phase HPLC.

### Trypsin digestion of protein

Casein (1picomole) was digested with trypsin using an enzyme to protein ratio of 1:50 in 25 mM ammonium bicarbonate buffer by incubating at 37 °C for 18 hours. The digest was dried on a speed vac concentrator and redissolved in 50% acetonitrile containing 0.1% trifluoroacetic acid before spotting on the MALDI target plate. The digest was also spiked with phosphopeptide (F16Kp) and spotted on the MALDI plate.

## Preparation of Samples for MALDI Analysis

Peptides were dissolved in water (5 picomoles/μl) and 1 μl of the sample was spotted on the MALDI target plate. The sample was allowed to air dry and 1 μl of matrix was spotted (5 mg/ml of 50% ACN containing 0.1% TFA) allowed it to air dry. The MALDI plate was inserted in to the mass spectrometer to acquire the spectra.

### MALDI TOF

A voyager DE-STR MALDI TOF mass spectrometer (Perceptive Biosystems, Framingham, MA, USA) was used for acquiring mass spectra in the positive ion reflector mode and linear mode. Negative ion spectra were also acquired. The mass spectrometer was fitted with a nitrogen laser (337 nm) for ionization. The laser-firing rate was 20 Hz. The grid voltage and delayed extraction time were optimized for obtaining good signals. The ion path length in linear and reflector mode is 2 meters and 3 meters respectively.

### MALDI TOF TOF

The mass spectra of the phosphopeptides and the casein digest with and with out spiking with the phosphopeptide were also acquired using a 4800 MALDI TOF TOF analyzer obtained from Applied Biosystems (Foster city, CA). The mass spectrometer was fitted with a Nd:YAG laser (355 nm) to ionize samples. The laser-firing rate was 200 Hz. The ion path length of linear, reflector and MS/MS modes are 1.5 meters, 3 meters and 2.4 meters respectively. It consists of a high-energy collision induced (CID) cell and spectra were obtained using air as CID gas with 1 KV and 2 KV energy in the positive ion mode. TOF TOF or tandem mass spectrometry consists of two successive TOF accelerations. In tandem mass spectrometry the first acceleration selects, isolate and fragment (collision with neutral gas) a precursor ion selected for that purpose. The second acceleration accelerates the precursor ion and fragments, and measures masses and intensities of fragment ions. The instrument is also capable of deflecting matrix ions and suppresses metastable ions.

## Results and Discussion

**Figure 1.**
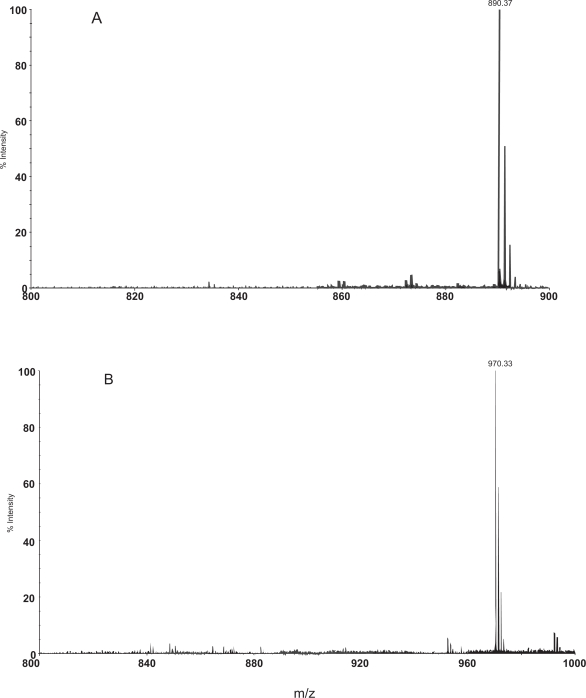
MALDI-TOF mass spectra of the peptides E8G (panel **A**) and E8Gp (panel **B**) recorded in the reflector mode using HCCA as matrix. The isotopic peaks of the peptides are also shown.

Several lines of evidence indicate that neutral loss of phosphate group occurs in phosphopeptides generated from ingel digest of proteins during the MALDI TOF acquisition of mass spectra in the reflector mode (Zhang et al. 1994; Zhang x et al. 1998). These peptides contain free amino group and carboxyl groups. In order to examine whether capping of the N and C termini modulates dephosphorylation during MALDI analysis, we examined the mass spectrum of E8G and E8Gp. The peptides with serine phosphorylated and non-phosphorylated stretching the region from residues 76 to 83 of the caveolin 1 (cav-1) were synthesized with capping of N and C terminals. Serine phosphorylation in this protein converts Cav-1 to a secretary protein from an integral membrane protein (Schelgel et al. 2001). The peptides exhibited molecular ion at m/z 890.37(Theoretical MH^+^ 890.36) and 970.33(Theoretical MH^+^ 970.33) respectively. The positive ion mass spectra of these peptides E8G and E8Gp recorded in the reflector mode of analysis are shown in Figures 1A and 1B. It is clear from Figure 1B that neutral loss of phosphate group was not observed in the reflector mode of spectrum acquisition with DE.

**Figure 2.**
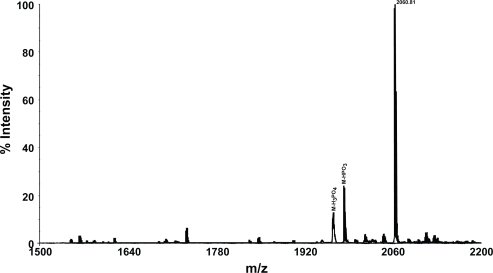
The MALDI TOF mass spectrum of the phosphopeptide F16Kp showing the molecular ion. Spectrum was recorded in the reflector mode. The peaks arising from the loss of phosphoric acid and phosphate moiety from the parent ion (M) are also shown.

The mass spectrum of the phosphopeptide F16Kp with free amino and carboxyl groups exhibited molecular ion at m/z 2061.81 (Theoretical MH^+^ 2061.82) (Fig. 2). The molecular ion of this peptide was observed with good intensity and without any neutral loss of phosphate group. Hence it appears that presence or absence of blocking group at the N- or C- terminal has no effect on the neutral loss of phosphate group. Form these studies it is clear that phosphopeptides cannot be distinguished from non-phosphorylated peptides from the peptide mass fingerprints used in the identification of proteins using MALDI TOF analysis with delayed extraction and in the reflector mode.

**Figure 3.**
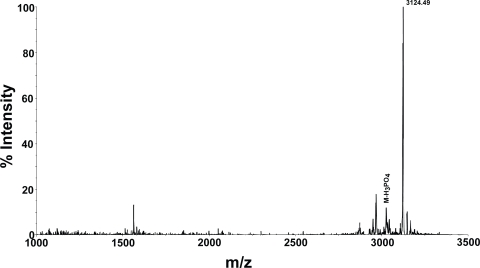
The MALDI TOF mass spectrum of the tetraphosphopeptide R25R recorded in the linear mode on Voyager DE STR MALDI TOF instrument. The loss of phosphoric acid from the parent ion (M) was indicated in the spectrum. The additional peaks are some impurities present in the sample.

In order to examine whether multiply phosphorylated peptides can be detected, the mass spectrum of a tetraphospho peptide from casein digest was examined. The molecular ion of this peptide could not be obtained in the reflector mode. In the linear mode, the molecular ion could be detected at m/z 3124.49 (Theoretical MH^+^ 3123.92) using Voyager DE STR mass spectrometer. The MALDI TOF mass spectrum of this peptide is shown in Figure 3. However, molecular ion for the tetra phosphopeptide could not be detected even in the linear mode of analysis with 4800 MALDI TOF TOF (data not shown).

**Figure 4.**
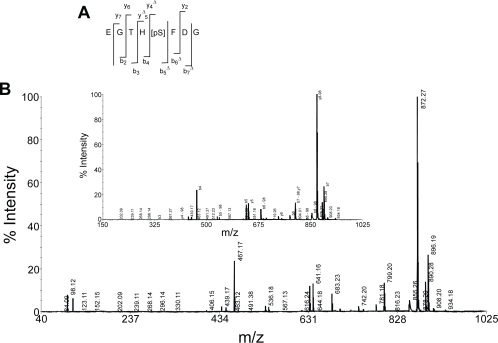
The CID mass spectrum of the phosphopeptide E8Gp recorded on 4800 MALDI TOF TOF mass analyzer. Panel **A** shows the fragmentation pattern of the peptide. Δ indicates the corresponding b_n_ or y_n_ ion minus H_3_PO_4_. Panel **B** shows the MS/MS spectrum of E8Gp. Number next to b_n_ or y_n_ ion indicates loss of H_3_PO_4_ .The inset shows expanded region of the spectrum showing b-type and y-type ions.

**Figure 5.**
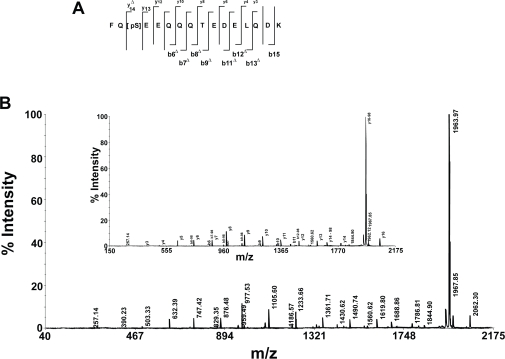
The CID mass spectrum of the monophospho peptide F16Kp recorded using 4800 MALDI TOF TOF mass analyzer. Panel **A** shows the fragmentation pattern of the peptide. Δ indicates the corresponding b_n_ or y_n_ ion minus H_3_PO_4_. Panel **B** shows the MS/MS spectrum of F16Kp. Number next to b_n_ or y_n_ ion indicates loss of H_3_PO_4_ .The inset shows some expanded region of the spectrum showing y-type and b-type ions.

In order to determine the stability of phosphate group during fragmentation, CID mass spectra of the peptidesE8Gp and F16Kp were recorded using 4800 MALDI TOF TOF mass analyzer using HCCA as matrix in the reflector mode. The results are shown in the Figures 4 and 5. The fragmentation of these peptides is shown in the inset of the mass spectra. The fragment ions of the peptides with loss of phosphate groups from y and b ions are shown in Table 1 and 2. Examination of the fragments of the b_n_ ion series indicates that the mass difference between b_5_ and b_4_ is 167 Da, corresponding to the b-ion of phosphoserine. Therefore, this establishes that in peptide E8Gp there is no loss of phosphate from phosphoserine. Further, the analysis of the b-type ions indicates the presence b_5_, b_6_ and b_7_ of the phosphoserine containing fragments at m/z 634.13, 781.18 and 896.19 respectively. b ions which correspond to loss of phosphate are also observed. These ions are identified at m/z 536.18, 683.23 and 798.24 that arise from ions b_5_ -98, b_6_ -98 and b_7_ -98 respectively (see inset of Fig. 4). Similarly, the y-type ions after phosphoserine residue y_4_, y_5_, y_6_ and y_7_ are observed at m/z 504.14, 641.16, 742.20 and 799.20 respectively. The ions corresponding to the loss of phosphate group from these ions i.e. y_4_-98, y_5_-98, y_7_-98 are observed at m/z 406.15, 543.20, and 701.25 respectively. The loss of phosphate group from y_6_ ion is not observed. Taken together, the fragmentation of b type and y type ions coupled with the fragments arising from the loss of phosphate group the location of the phosphoserine could be localized at residue 5. The b-type ions are more intense as compared to the y-type ions (Table 1, Fig. 4). A similar analysis of F16Kp indicates the location of the phosphoserine at residue 3. Figure 5 shows the MS/MS spectrum of F16Kp and Table 2 shows the characteristic peaks for the identification of the sequence of the peptide. Analysis of y_n_ series of ions of F16Kp reveals that the mass difference between y_14_ at m/z 1619.80 and y_13_ at m/z 1786.81 is 167 Da corresponding to the y ion of phosphoserine. Loss of H_3_PO_4_ from the molecular ion appeared at m/z 1963.97 and it is the most intense peak in the spectrum. This suggests the presence of a phosphoamino acid in the peptide. Loss of phosphate moiety from y_14_ ion detected at m/z 1688.86. A series of b ions of the peptide are observed at m/z 829.35, 957.46, 1085.50, 1186.57, 1315.59, 1430.62, 1559.30 and 1673.38 corresponding to b_6_ to b_13_ respectively. Loss of phosphate group from these fragments is also found at m/z 731.36, 859.44, 981.51, 1088.58, 1217.62, 1332.64, 1461.72 and 1574.77. The intensity of y_n_ series of ions is more as compared to the b_n_ series of ions (Table 2, Fig. 5). Thus, form these fragments the phosphorylation site in the peptides could be detected.

**Figure 6.**
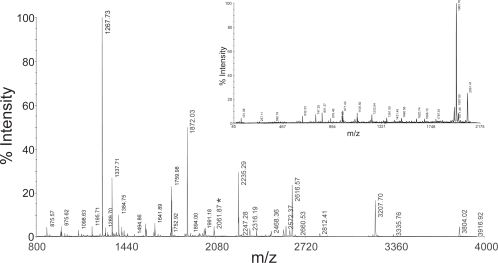
The trypsin digest of the protein casein spiked with the phosphopeptide. The inset shows the CID mass spectrum of the monophosphopeptide. The symbol (*) in the mass spectrum indicates the spiked peptide.

Trypsin digest of Casein with and with out spiking with the phosphopeptide (F16Kp) was also analyzed. Figure 6 shows the PMF of the casein digest spiked with the phosphopeptide. The protein could be identified upon searching with database and the CID mass spectrum of phosphorylated peptide was shown in the inset Figure 6, which was same as the MS/MS of the purified phosphopeptide. This shows that the phosphopeptides could be identified form the peptide mass fingure print of the protein.

In conclusion, neutral loss of phosphate group was not observed in the mass spectrum of serine phosphorylated monophospho peptides in the reflector mode of analysis using either voyager DE STR MALDI TOF or 4800 MALDI TOF TOF mass spectrometers. The earlier analysis of phosphopeptides using MALDI TOF mass spectrometers contain single stage acceleration regions and have no control over the voltages that define the initial acceleration region and the fragmentation using different mass spectrometers might be different. However, introduction of delayed extraction (DE) in these mass spectrometers greatly helps in controlling the side chain fragmentation, improves mass accuracy and resolution (Juhasz et al. 1997; Vestal et al. 1995). The molecular ion of a tetra phosphate peptide could be detected in linear mode of analysis using Voyager MALDI TOF mass spectrometer. Low laser pulse rate (3 or 20 Hz), higher laser energy (337 nm) might have played a role in detecting the molecular ion of the tetra phosphopeptide in the Voyager DE STR MALDI TOF MS.

The phosphorylation sites could be detected with the help of CID MS spectra recorded with MALDI TOF TOF mass analyzer by direct observation of phosphorylated b and y ions in addition to their dephosphorylated counterparts. Thus, the instrumental design and the experimental conditions play an important role in the analysis of phosphopeptides.

## Figures and Tables

**Table 1.** Characteristic daughter ions obtained from MS/MS of the phosphopeptide E8Gp.

Residue*	**b**	**Intensity**	**b-H_3_PO_4_**	**Intensity**	**y**	**Intensity**	**Y-H_3_PO_4_**	**Intensity**
E	-	-	-	-	928.19	157	-	
G	-	-	-	-	799.20	5243	701.25	569
T	330.11	169	-	-	742.20	1015	-	-
H	467.16	9200	-	-	641.16	5076	543.20	853
[pS]	634.13	4649	536.18	1053	504.14	77	406.15	286
F	781.18	1317	683.23	3271	-	-	-	
D	896.19	10000	798.24	1027	-	-	-	
G		-	855.26	2058	-	-	-	

* [pS] indicated the phosphoserine residue.

**Table 2.** Characteristic daughter ions obtained from MS/MS of the phosphopeptide F16Kp.

Residue*	**b**	**Intensity**	**b-H_3_PO_4_**	**Intensity**	**y**	**Intensity**	**y-H_3_PO_4_**	**Intensity**
F	-	-	-	-	-	-	1963.52	76000
Q	-	-	-	-	1914.70	913	1817.90	982
[pS]	-	-	-	-	1786.81	1825	1688.48	2407
E	-	-	474.07	197	1619.80	3272	-	-
E	701.21	240	603.07	258	1490.72	3184	-	-
Q	829.35	506	731.34	358	1361.71	3930	-	-
Q	957.46	842	859.44	1443	1233.66	5914	-	-
Q	1085.50	932	987.51	1216	1105.60	6820	-	-
T	1186.57	618	1088.58	2033	977.53	8903	-	-
E	1315.59	732	1217.62	1716	876.48	3832	-	-
D	1430.62	1490	1332.64	1394	747.42	3670	-	-
E	1559.30	763	1461.72	985	632.39	3402	-	-
L	1673.38	111	1574.77	881	503.33	775	-	-
Q	1800.00	605	-	-	390.23	324	-	-
D	1915.39	913	1817.90	982	-	-	-	-
K	-	-	1945.99	3805	-	-	-	-

* [pS] indicated the phosphoserine residue.
